# New Insights into TFEB SUMOylation and Its Role in Lipid Metabolism and Cardiovascular Disease

**DOI:** 10.3390/ijms27010347

**Published:** 2025-12-29

**Authors:** Qingxiu Meng, Chun Guang Li, Xiaolong Chen, Rui Cao, Haihong Zhang, Ping Wang, Jing Jin

**Affiliations:** 1 School of Physical Education, Taiyuan University of Science and Technology, Taiyuan 030024, China; mengqingxiu@tyust.edu.cn; 2 NICM Health Research Institute, Western Sydney University, Westmead NSW 2145, Australia; c.li@westernsydney.edu.au (C.G.L.); 20130007@hznu.edu.cn (P.W.); 3 School of Physical Education, Hangzhou Normal University, Hangzhou 311121, China; 2022111005013@stu.hznu.edu.cn (X.C.); 20070062@hznu.edu.cn (H.Z.)

**Keywords:** SUMOylation, transcription factor EB, lipid metabolism, cardiovascular diseases

## Abstract

Transcription factor EB (TFEB) plays a crucial role in lipid metabolism and is indispensable for maintaining intracellular metabolic homeostasis. Its functionality relies significantly on its subcellular localization and transcriptional activity. Recent studies have revealed that SUMOylation regulates the subcellular localization and transcriptional activity of TFEB. Numerous studies indicate that mutations or dysfunctions of TFEB SUMOylation sites, as vital regulatory mechanisms, are closely associated with lipid metabolism in cardiovascular disease. Thus, in this review, we provide an overview of the current knowledge and recent advances in TFEB SUMOylation, with a particular focus on the mechanism of TFEB SUMOylation and its role in lipid metabolism, providing potential new strategies for developing novel therapeutic treatments for cardiovascular diseases.

## 1. Introduction

Cardiovascular disease is a common aliment, increasingly affecting younger populations and exhibiting a low control rate [1]. Clinically, complications associated with cardiovascular disease are linked to prolonged abnormalities in lipid metabolism, especially hypercholesterolemia, which leads to endothelial cell damage and the formation and accumulation of foam cells in the vascular wall, ultimately resulting in plaque formation [2]. At present, it remains unclear whether this outcome represents a primary defect or a secondary event. However, existing research has suggested a potential connection between these pathological changes and the translocation and activity of transcription factors [3], sparking research interest in molecularly targeted therapies for cardiovascular diseases. Transcription factor EB (TFEB), which regulates major proteins and molecules in signaling pathways through nuclear translocation, is considered a potential target for the development of new therapies for cardiovascular diseases [4]. It serves as a key link across various signaling pathways, including lipid metabolism, cell aging, DNA repair, and endoplasmic reticulum stress [5]. For example, studies in Apoe knock-out mice with a high-fat diet found that TFEB was accumulated in the cytoplasm, resulting in a significant reduction in its activity and exacerbating atherosclerosis, whereas, curcumin promoted lipid degradation by increasing TFEB nuclear translocation and alleviating atherosclerosis [6]. Additionally, Zhao et al. reported that TFEB siRNA in mouse macrophage foam cells significantly increased lipid accumulation, ultimately contributing to the development of atherosclerosis [7].

Further studies have shown that TFEB knockdown (shTFEB) in endothelial cells increased cholesterol synthesis and inhibited vascular development and angiogenic remodeling [8]. Therefore, disturbances or decreases in TFEB activity can lead to the onset and progression of various cardiovascular diseases [9]. Among the studies investigating TFEB activity, the most scrutinized aspect is the post-translational modification of proteins. To date, several post-translational modifications have been identified, including phosphorylation, ubiquitination, methylation, and SUMOylation [10]. SUMOylation is a recently discovered and crucial post-translational modification mechanism that regulates the activity and subcellular localization of TFEB [11]. SUMOylation has been shown to exert a complex effect on TFEB activity, thereby influencing lipid metabolism in cardiovascular disease [12]. Therefore, this review highlights recent research findings on the regulation of TFEB SUMOylation in lipid metabolism and its related molecular mechanisms to provide insights and a theoretical basis for developing potential novel therapeutics for related cardiovascular diseases.

## 2. Structure and Function of TFEB

**Figure 1:**
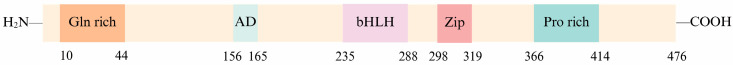
Structures of TFEB protein [9,16]. Abbreviations: Gln: glutamine rich region; AD: activation domain; bHLH: basic helix–loop–helix; Zip: leucine zipper region; Pro: proline-rich region. Created in BioRender (Xiaolong, C. (2025) https://app.biorender.com/illustrations/6910937ea4e6e03c414a94c8).

TFEB is a transcriptional regulator of the microphthalmia-associated transcription (MIT) factor family, consisting of 476 amino acids and three different domains [13]: (1) a basic helix–loop–helix leucine zipper (bHLH-Zip) structure, serving as the alkaline DNA binding domain; (2) an acidic transcription activation domain and a glutamic-acid-rich domain, functioning as a transcription activation region; (3) a serine-rich domain and other domains [13]. TFEB can recognize not only palindromic CACGTG E-box sequences, but also asymmetric TCATGTG M-box sequences which are not recognized by other bHLH-Zip transcription factors [14]. Moreover, TFEB forms dimmers with homologous or heterogenic deoxyribonucleic acid (DNA) groups to initiate transcription of the target genes [15] (Figure 1).

## 3. Process of SUMOylation

**Figure 2:**
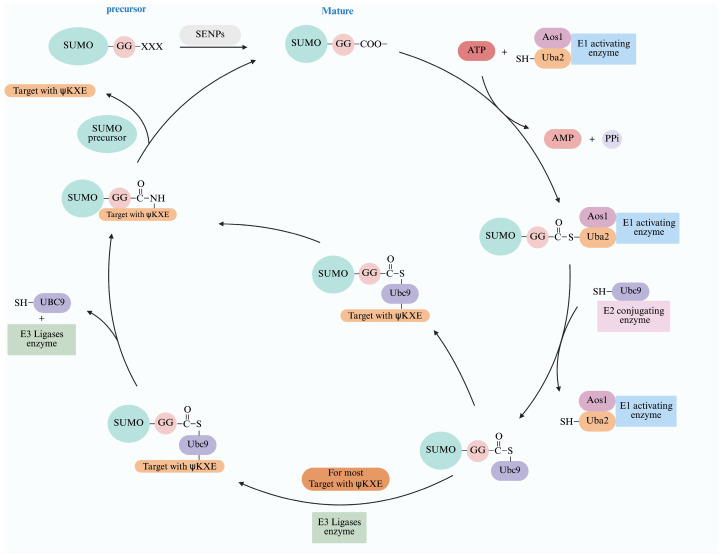
An overview of the cascade process of SUMOylation adopted from [17,26,27]. After SUMO gene transcription and translation, an immature SUMO protein is formed, which matures into SUMO GG under the action of SENPs and then undergoes SUMOylation under the action of a series of enzymes, including the E1 activating enzyme, E2 conjugating enzyme and the E3 ligases. Meanwhile, SUMO-target proteins remove SUMO by SENPs, allowing them to engage in a new cycle. Abbreviations: Gly: G; the E1 activating enzyme that consists of two subunits, Aos1 and Uba2; the E2 conjugating enzyme: Ubc9; the E3 ligation enzyme, which currently has three different types, RanBP2, PIAS, and Pc2. Created in BioRender (Xiaolong, C. (2025) https://app.biorender.com/illustrations/685263b479cff248256fb45f).

SUMOylation is an evolutionarily conserved post-translational modification characterized by a series of enzymatic reactions that covalently bind the carboxylic terminal of the SUMO protein to the lysine residues of the target protein [17]. Proteins encoded by the SUMO gene contain a short peptide chain at the C-terminus known as a SUMO precursor protein, which inhibits SUMO activity. These carboxyl (C)-terminal peptide chains are cleaved by SUMO-specific carboxyl-terminal hydrolase (SENPs/Ulps), exposing the C-terminal diglycine (glycine–glycine) motif. This process activates the SUMO protein and is commonly referred to as “SUMO maturation” [18]. The mature SUMO protein is covalently attached via a thioester bond between the C-terminal Gly residue and a cysteine (Cys residue) in the catalytic subunit Uba2/Aos1 of the heterodimeric SUMO E1-activating enzyme (E1) in an ATP-dependent reaction [18]. The activated SUMO protein is subsequently transferred from the E1 to the Ubc9 (SUMO protein binding enzyme Ubc9, E2), which recognizes and attaches SUMO to a lysine residue on the target protein, typically though not exclusively, at canonical consensus motifs [17]. Finally, polypeptides known as SUMO ligation enzymes (E3s), such as PIAS, RanBP2, and Pc2, play a crucial role in facilitating SUMOylation by interacting with both Ubc9 and the target protein, acting as bridging factors to enhance the efficiency of the SUMO conjugation for many, though not all, target proteins [17,19]. These enzymatic reactions result in the formation of multi-and/or poly-SUMOylation. Meanwhile, SENPs remove SUMO from target proteins via C-terminal hydrolase and isopeptidase activities allowing SUMO to engage in a new cycle [20] (Figure 2), but their precise mechanism is not completely understood (Figure 2).

Interestingly, the SUMOylation process is dynamic and reversible. SUMO-modified proteins can be deSUMOylated by SENPs [17,21], which features a conserved C-terminus catalytic domain that catalyzes the dissociation of SUMO from target proteins [22] (Figure 2). The currently known SENPs consists of six members: SENP1, SENP2, SENP3, SENP5, SENP6, and SENP7 [22]. Different SENPs selectively act on distinct SUMO-conjugated proteins [22]. SENP1 and SENP2 catalyze deSUMOylation for all SUMO isoforms, whereas SENP3, SENP5, SENP6, and SENP7 preferentially target SUMO2/3-conjugated proteins [22,23,24,25]. Maintaining the dynamic balance during SUMOylation is critical for normal cellular functions and disease pathogenesis.

## 4. SUMOylation of TFEB

**Figure 3:**
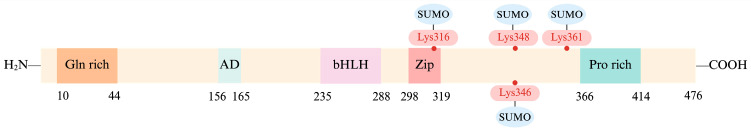
SUMO1ylation sites of TFEB. TFEB’s transcriptional activity is regulated by SUMOylation. However, the site of SUMOylation varies among different species. Abbreviations: Gln: glutamine rich region; AD: activation domain; bHLH: basic helix–loop–helix; Zip: leucine zipper region; Pro: proline rich region; K: Lysine. Created in BioRender (Xiaolong, C. (2025) https://app.biorender.com/illustrations/69133de769a89c9337a780d5).

Recent studies have found that the transcriptional activity of TFEB is mainly driven by SUMOylation [26]. In mammals, the identified SUMO proteins include SUMO1, SUMO2, and SUMO3 [25]. TFEB can only be SUMOylated by SUMO1. However, the lysine sites targeted for SUMO1 conjugation vary across cell types. For example, the SUMO1ylation site of TFEB is lysine 346 in mouse bone marrow macrophages, lysine 316 in COS-7 cells, lysine 348 in mice myocardium and lysine 361 or 316 in human bone marrow macrophages [11,12,27] (Figure 3). It is noteworthy that the lysine residues of the TFEB protein that undergo SUMO1ylation conform to the conserved consensus sequence ΨKXE (Ψ representing a hydrophobic amino acid, usually valine, leucine, or isoleucine; K representing lysine which serves as the SUMOs binding site; X representing any amino acid; E representing glutamic acid) [28]. Abnormal SUMOylation may cause changes in subcellular localization and the stability and transcriptional activity of TFEB proteins [29], ultimately leading to cardiovascular disease, metabolic disease, neurodegenerative disease, and tumors [18]. However, how multi- and/or poly-SUMOylation affects TFEB’s subcellular distribution, transcriptional activity, and protein-to-protein interactions remains to be further studied.

## 5. Biological Effects and Subcellular Localization of TFEB SUMOylation

**Figure 4:**
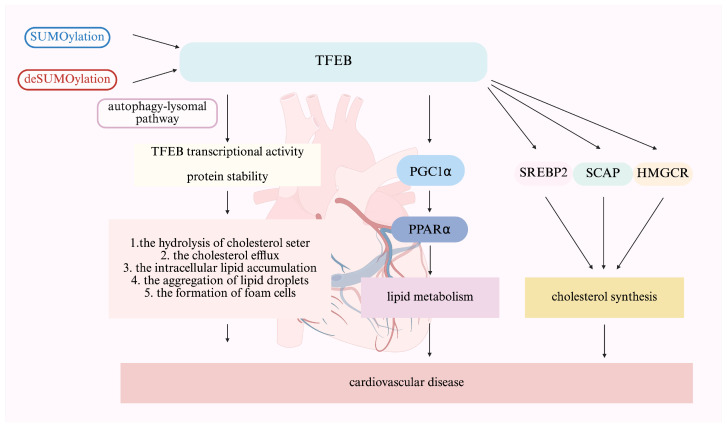
TFEB SUMOylation targeting for regulating lipid metabolism in cardiovascular disease. TFEB SUMOylation can simultaneously directionally regulate lipid metabolism network dynamics, including lipid droplets, cholesterol efflux, cholesterol synthesis, and lipid catabolism. Abbreviation: Peroxisome proliferator-activated receptor gamma coactivator-1 alpha (PGC1a); Peroxisome proliferator-activated receptor alpha (PPARa); Sterol regulatory element-binding protein 2 (SREBP-2); SREBP-lysis-activated protein Sterol sensor (SCAP); 3-hydroxy-3-methylglutaryl-CoA reductase (HMGCR). Created in BioRender (Xiaolong, C. (2025) https://app.biorender.com/illustrations/69109b54c9ab00f66d8b45bf).

Proteins modified by SUMOylation can undergo changes in their activity and their protein–protein interaction, thereby affecting protein degradation and function [19,30]. For example, Zhang Le [27] showed that TFEB SUMOylation increased its transcriptional activity, inhibited its degradation, maintained its protein stability, and promoted the transcription level of its target genes, representing a new mechanism of protection against myocardial injury in aging ischemia and reperfusion. Similarly, Pang Q’ [12] also showed that TFEB SUMOylation promoted its transcriptional activity. In contrast, Wang KZ [11,31] found that TFEB SUMOylation inhibited its transcriptional activity. To better understand this discrepancy, Wang KZ [11] analyzed the structure of TFEB and found that the SUMOylation site was located near the DNA-binding domain of TFEB, thereby inhibiting its transcriptional activity by weakening the binding ability of TFEB to target genes. However, the exact mechanism is still not fully understood and may involve the inhibition of TFEB’s interactions with co-activators, enhanced binding with co-repressor factors, or regulation of the transcriptional activity of TFEB through other signaling pathways [11]. In addition, Miller AJ [32] reported that, although TFEB SUMOylation decreased its transcriptional activity, it does not alter its DNA binding, stability, or nuclear localization. Therefore, Miller AJ [32] proposed the questions “Is SUMOylation of TFEB a constitutive process or a regulated one?” and “If regulated, what signals trigger or inhibit the pathway?” It is possible that the decreased transcriptional activity may be due to an alteration in protein–protein interaction by a promoter with multiple TFEB binding sites. It has been previously suggested that SUMO can regulate transcription by recruiting repressive elements to promoters [33]. TFEB SUMOylation is also involved in chromosome formation and the regulation of DNA repairs [13,34,35] (Figure 4). The reasons for the inconsistency among the above research findings regarding the transcriptional activity of SUMOylated TFEB may include the following: (1) TFEB contains multiple potential SUMOylation sites, some of which can recruit transcriptional co-repressors containing SUMO-interacting motifs (which specifically recognize and bind SUMO proteins), such as HDAC1, or directly interfere with the interaction between SUMOylation sites and transcriptional co-activators (e.g., CBP/p300), thereby inhibiting TFEB transcriptional activity [32]; Other SUMOylation sites are located near the nuclear localization signal of TFEB, counteracting TFEB’s nuclear export signal or enhancing its binding to nuclear scaffold proteins, thereby stabilizing TFEB in the nucleus and increasing its transcriptional activity [36,37]. Thus, different SUMOylation sites mediate distinct biological responses; (2) SUMOylation of TFEB is influenced by environmental factors such as oxidative stress and nutritional status, leading to variations in its transcriptional activity [38,39]; (3) SUMOylation is also regulated by upstream post-translational modifications, including phosphorylation, acetylation, or ubiquitination, which in turn affect the transcriptional activity of TFEB [24]. Overall, although TFEB preserves one of the consensus sequences, the specific effect of SUMOylation on TFEB activity and the cellular contexts that regulate TFEB SUMOylation remain to be determined [32].

Apart from regulating the transcriptional activity and function of TFEB through SUMOylation, the effect of subcellular localization and modification sites of TFEB has also attracted increasing attention. Notably, TFEB is primarily located in the cytoplasm under normal cellular conditions. In response to stress, it translocates into the nucleus to promote organismal homeostasis, However, research on nuclear translocation mainly focuses on phosphorylation modification. It remains unclear whether the subcellular localization of TFEB changes upon SUMOylation and whether the modified sites are consistent across different contexts. Zhang Le [27] demonstrated that TFEB SUMOylation occurs in the nucleus and identified lysine 348 of TFEB as the SUMOylation site in cardiomyocytes using myocardial hypoxia/reoxygenation and ischemia/reperfusion mouse models. Moreover, Miller AJ reported that lysine 316 is the SUMOylation site in B16 melanoma, COS7, and HEK293 cells [32]. Although the subcellular location of TFEB SUMOylation was not directly reported in Miller AJ’s study, indirect evidence suggests that it also occurs in the nucleus, as their findings clearly indicate that SUMOylation inhibits TFEB transcriptional activity through histone deacetylases 1 (HDAC1), a typical nuclear protein located in the chromatin of the cell nucleus [32]. Pang Q [12] also identified lysine 316 as the SUMOylation site of TFEB and the difference is that both GFP-TFEB and GFP-TFEB K316R (in which lysine 316 was mutated to arginine) were dispersed in the nucleus and cytoplasm in HeLa cells transfected with GFP-TFEB and GFP-TFEB K316R, respectively, under normal conditions. After 3 h starvation, both GFP-TFEB and GFP-TFEB K316R accumulated in the nucleus. This finding, confirmed through cytoplasmic and nuclear experiments, suggests that TFEB SUMOylation occurs in both compartments and that SUMOylation does not alter the subcellular localization of TFEB. Similarly, Wang KZ [11] found that TFEB SUMOylation did not affect TFEB’s subcellular localization in HEK293T cells transfected with GFP-TFEB and GFP-TFEB K316R and incubated with chloroquine (50 μg/mL). However, Wang KZ [11] found that lysine 361 was the only site in human cells and lysine 346, rather than 316, was the only SUMOylation site in murine cells. These findings suggest that TFEB SUMOylation does not consistently influence TFEB nuclear translocation, and the modification sites of TFEB SUMOylation may vary. Several factors may explain these differences: (1) The different amino acid sequences of TFEB among different species; (2) Environmental factors such as intracellular nutritional status that may also affect the SUMOylation site of TFEB; (3) E3 ligase. However, since different E3 ligases may recognize different sites of TFEB, the specific E3 ligases for TFEB SUMOylation have not been fully clarified at present. Determining these ligases may help further explain the variability in modification sites. (4) TFEB may have co-occurring post-translational modifications, such as phosphorylation modifications, and these modifications may affect the SUMOylation site selection.

Importantly, although most SUMO conjugates are found in the nucleus, SUMO proteins and the enzymes involved in the SUMOylation pathway are present in both the nucleus and the cytoplasm [40]. However, the mechanism by which SUMOs and associated enzymes translocate from the cytoplasm into the nucleus remains unclear. Interestingly, SENP1 (SUMO-specific protease 1), a key member of the SENPs family, plays an essential role in maintaining TFEB SUMOylation homeostasis. Its primary role is to cleave SUMO1 protein from TFEB and it has been confirmed that SUMO1ylation of TFEB is regulated by SENP1 [41]. Moreover, SENP1 is also crucial for TFEB deSUMOylation [41]. Additionally, the reduction of SUMOylation in mitochondria occurs only in the presence of SENP1. Silencing the SENP1 gene resulted in the disappearance of SUMOylation, indicating the indispensable role of SENP1 in mitochondria SUMOylation [42]. Thus, the dynamic interplay of SUMOylation and deSUMOylation is finely regulated and ultimately influences TFEB’s biological function. However, at present, the mechanisms by which SENP1 regulate the subcellular localization, stability, transcriptional activity, and protein–protein interaction of TFEB have not been reported. In addition, the role of deSUMOylation in modulating TFEB through cytoplasm–nucleus trafficking remains poorly understood. Existing studies have shown that mutation of a phosphorylation site of SENP3 (a subtype of human SENPs) results in increased deSUMOylation function during mitosis, which leads to impaired SUMOylation of various chromosome-binding proteins and ultimately affects chromosome stability [43]. These findings suggest that deSUMOylation also plays a crucial role in the SUMOylation process, possibly through promoting phosphorylation or interacting with other regulatory mechanisms.

SUMOylation, as a widespread and important post-translational protein modification in cells, can influence numerous cellular processes, including transcriptional regulation and intracellular signal transduction, to ensure normal cell division and maintain genome stability and integrity [44,45,46]. It is also involved in various physiological and pathological processes such as the prevention of cardiovascular disease, the treatment of heart failure, tumor metastasis, and adipocyte differentiation [47,48,49]. During these processes, SUMOylation can switch a protein’s function from a transcriptional activator to a transcriptional repressor, representing a novel mechanism relevant to various diseases. Thus, balanced phosphorylation, SUMOylation, and deSUMOylation of TFEB are essential for the precise transcriptional control of its target genes. Further investigations are required to gain a comprehensive understanding of the distinct mechanisms underlying SUMOylation and deSUMOylation. Further research will likely focus on how SUMOylated TFEB regulates the cellular localization of downstream proteins, the activity of related enzymes, and protein–protein interactions, ultimately influencing various cellular functions.

## 6. Roles of TFEB SUMOylation in Regulating Lipid Metabolism

TFEB plays a crucial role in regulating lipid metabolism (Figure 4). Recent studies have demonstrated that TFEB overexpression significantly upregulates the expression of the peroxisome proliferator-activated receptor gamma coactivator 1α (PGC1α) gene, a key regulator of hepatic lipid metabolism. This occurs through TFEB direct binding to two CLEAR sites on the PGC1α gene and subsequently regulates lipid metabolism via its downstream nuclear receptor, peroxisome proliferator activated receptor α (PPARα). Activation of PPARα in liver tissue is essential for TFEB-induced gene transcription, as shown by microarray analysis [50]. Additionally, TFEB is involved in intracellular lysosomal lipid-solubility-mediated lipid phagocytosis [9]. This mechanism involves TFEB specifically recognizing and binding to the upstream cis-acting element CLEAR box (5′-GTCACGTGAC-3′) of the autophagy/lysosomal biogenesis-related genes, thereby upregulating lipid degradation and efflux [10]. In addition, TFEB regulates cholesterol synthesis by controlling the transcription of genes encoding key regulatory factors such as sterol regulatory element-binding protein 2 (SREBP-2), the SREBP-lysis-activated protein Sterol sensor (SCAP), and the SREBP-2 target gene HMGCR (β-hydroxy-β-methylglutaryl-CoA reductase) via binding to the CLEAR motif in the promoter region of these target genes [50,51,52,53]. These mechanisms collectively contribute to relieving the progression of atherosclerosis and stabilizing plaque [9]. However, the intricate regulatory network controlling TFEB activity and function remains incompletely understood. Recent studies have shown that TFEB SUMOylation can affect both its activity and downstream metabolic functions.

TFEB SUMOylation has been found to regulate cholesterol efflux and inhibit the formation of macrophage foam cells. Using the SUMO-site-mutated TFEB (TFEB K316R) in a human THP-1 macrophage cell line, it was found that SUMOylation of TFEB promoted the hydrolysis of cholesterol ester in macrophages, increased cholesterol efflux, reduced intracellular lipid accumulation, and inhibited the aggregation of lipid droplets and the formation of foam cells within macrophages by promoting lysosome production and autophagy activation [42]. Notably, the increased cholesterol efflux was observed only in cholesterol efflux mediated by apoA-I and there was no corresponding change in cholesterol efflux mediated by HDL. This finding is consistent with the observation by [54] that autophagy mainly inhibits cellular lipid accumulation by promoting ApoA-I-mediated cholesterol effusion in macrophages. Thus, cholesterol efflux can be regulated by modulating lysosome and autophagy activity through the SUMOylation mechanism of TFEB. In contrast, Wang KZ [11,31] reported that the deSUMOylation of TFEB enhanced the transcriptional activity of TFEB, increased lysosomal production and autophagy, and inhibited the formation of macrophage foam cells, resulting in subsequently decreased lipid deposition in bone marrow-derived macrophages and C57BL/6 mice, ultimately inhibiting the development of atherosclerosis. Interestingly, TFEB SUMOylation did not affect the expressions of the cholesterol uptake and efflux-related receptors such as the cluster of differentiation 36 (CD36), scavenger receptor class B member 1 (Scarb1), macrophages scavenger receptor 1 (Msr1) and ATP-binging cassette G1 (Abcg1), nor the uptake of low-density lipoprotein (LDL) [31]. These findings suggest that the TFEB SUMOylation mechanism may not be the sole mechanism that regulates the metabolism of cholesterol and lipid in macrophages and additional molecular mechanisms remain to be elucidated. In fact, in addition to cholesterol metabolism, lipolysis-dependent autophagy and neutral lipolysis are also important for maintaining lipid flux homeostasis [55]. These processes may also be regulated by TFEB SUMOylation (Figure 4). As a key transcription factor for autophagy and lysosomes, TFEB has been shown to modulate intracellular lipid degradation and efflux by regulating the nuclear translocation of lipophagy-associated genes [56]. However, how TFEB regulates lipolysis-dependent autophagy and neutral lipolysis after SUMOylation remains unknown and warrants further investigation.

In addition, SENP1, which catalyzed deSUMOylation of TFEB, has also been implicated for regulating the dynamics of lipid metabolism. Knockout of SENP1 in macrophages decreased lipid accumulation even under stimulation by oxidized low-density lipoprotein [57]. Metabolomics analysis of cholesterol metabolites showed that 10 of the 15 metabolites in cholesterol metabolism were significantly decreased in macrophages with SENP1 knocked out, suggesting that SENP1 may regulate the excretion of cholesterol metabolites by deSUMOylation of TFEB [41]. It has also been shown that SENP1 knockout increased the autophagy activity in macrophages, especially lipid phagocytosis, and enhanced the ability of macrophages to process intake of lipoproteins, thereby preventing lipid accumulation and foam cell formation [41]. Thus, SENP1-mediated regulation of TFEB SUMOylation may be a potential driver of peripheral vascular degeneration associated with lipid metabolic disorders. At present, although SUMOylation and deSUMOylation are recognized as primary pathway regulating TFEB activity, their underlying mechanisms remain incompletely understood. For example, it is still unclear how specific sites of SUMOylation and deSUMOylation regulate TFEB function, how TFEB is diverted to the cytoplasm after entering the nucleus, and how the nuclear export of TFEB is regulated. Further research is needed to elucidate these mechanisms.

Recent evidence highlights that TFEB is highly conserved in its ability to link autophagy to lipid homeostasis and longevity across various species, including *Caenorhabditis elegans* (*C. elegans*) [58], murine models [59], and human cells [60]. Moreover, different types of fatty acids and the duration of exposure to exogenous fatty acids have been shown to have different effects on TFEB content in murine cardiomyocytes. Palmitate, a saturated fatty acid, decreased TFEB levels in a time- and concentration-dependent manner, whereas polyunsaturated fatty acids had no effect [61], further highlighting the crosstalk between TFEB and lipid metabolism homeostasis. In mice overexpressing TFEB via an adenoviral vector that expresses human TFEB (HDAd-TFEB), lipid metabolism is upregulated through the induction of the peroxisome-proliferator-activated receptor γ coactivator 1 α (PGC1α) [50]. Conversely, *Tfeb*-KO mice exhibited an accumulation of lipid droplets and higher levels of circulating free fatty acids and glycerol. These findings indicate that TFEB plays a central role in regulating cellular lipid metabolism [50]. However, further research is needed on the targeted regulation of lipid metabolism by TFEB SUMOylation.

## 7. Roles for TFEB SUMOylation in Cardiovascular Diseases

TFEB SUMOylation has been found to play an essential role in maintaining cardiovascular function homeostasis by regulating lipid metabolism. TFEB SUMOylation may be a potential mechanistic pathway contributing to cardiovascular function. Indeed, recent studies have linked TFEB SUMOylation with cardiovascular disease.

Atherosclerosis is a common cardiovascular disease and its typical early pathological changes involve the formation of macrophage foam cells [62]. Because cellular cholesterol metabolism is involved in the formation of macrophage foam cells, reduced cholesterol efflux can lead to lipid accumulation and subsequent foam-cell development, which is the primary mechanism underlying atherosclerosis [63,64,65]. Therefore, reducing macrophage foam-cell formation is central to treating atherosclerosis and related diseases. Recent studies have shown that TFEB is a key regulatory factor affecting lipid metabolism [38]. For example, TFEB overexpression has been shown to increase the expression of genes involved in lipid metabolism, including lipid and fatty acid catabolism, fatty acid binding and transport, fatty acid oxidation, sphingoid catabolism, and steroid catabolism [59,60]. Thus, TFEB is considered a protective factor against the occurrence and development of atherosclerosis. It has been demonstrated that TFEB SUMOylation in THP-1 cells increased its transcriptional activity, promoted the expression of downstream autolysosome-related genes, enhanced autolysosome formation and the autophagy process, increased the hydrolysis of cholesterol esters in macrophages, elevated apoA-I–mediated cholesterol efflux, and reduced intracellular cholesterol ester accumulation. However, it did not affect HDL-mediated cholesterol efflux, suggesting that TFEB SUMOylation can promote apoA-I–mediated cholesterol efflux in macrophages through autophagy. This process reduces cholesterol ester accumulation in macrophages, decreases foam cell formation, and ultimately inhibits the development of atherosclerosis [12]. In addition, TFEB deSUMOylation in TFEB-K346R *Ldl*^−^/^−^ mice and bone marrow–derived macrophages (BMDMs) increased TFEB transcriptional activity, enhanced lysosomal activity, increased the number of perinuclear lysosomes, and improved lysosomal degradative capacity. This deSUMOylation reduced the levels of IL-1β (a key pro-inflammatory cytokine and central inflammatory mediator linking lipid accumulation, immune responses, and plaque instability), increased the expression of cholesterol efflux receptors, enhanced cholesterol efflux, inhibited macrophage foam cell formation, alleviated lipid deposition, and ultimately suppressed the initiation and progression of atherosclerosis [11]. Conversely, TFEB SUMOylation has also been reported to inhibit cholesterol efflux in macrophages by weakening TFEB binding to its target genes and suppressing its transcriptional activity, lysosomal biogenesis, and cellular autophagy. These effects lead to enhanced inflammatory responses, increased lipid deposition, thickened vascular plaques, and accelerated atherosclerosis in mammalian cell lines and atherosclerotic mice [11]. The underlying mechanism may involve key inflammatory factors, such as oxidized low-density lipoprotein (ox-LDL), which activate specific SUMO E3 ligases in macrophages. These enzymes catalyze the SUMOylation of TFEB at specific lysine residues, thereby promoting TFEB binding to 14-3-3 proteins in the cytoplasm or allowing TFEB to remain in the cytoplasm and interfering with its nuclear localization signal and transcriptional activity [31,66]. Even when a small fraction of TFEB enters the nucleus, SUMOylation can recruit transcriptional co-repressor complexes containing SUMO-interacting motif (SIM) domains, such as HDAC1 and HDAC2, preventing TFEB from effectively initiating gene transcription [31,67,68]. As a consequence of TFEB inactivation, expression of downstream genes involved in lysosomal biogenesis and autophagy is reduced, thereby impairing lysosomal formation and autophagic activity. Furthermore, fewer lysosomes and reduced acidic hydrolase activity could impair the efficient hydrolysis of cholesterol esters to free cholesterol, thereby diminishing apoA-I–mediated cholesterol efflux and inducing cholesterol metabolic dysfunction. Meanwhile, TFEB inactivation can indirectly suppress the expression of the cholesterol efflux transporter ATP-binding cassette transporter A1 (ABCA1), further obstructing cholesterol efflux. Consequently, unhydrolyzed cholesterol esters accumulate in the cytoplasm as large lipid droplets, driving macrophage transformation into foam cells—the hallmark cells of atherosclerotic plaque formation [31]. However, the exact molecular mechanism of TFEB SUMOylation in cholesterol transport and excretion remains to be elucidated. Hence, further research is still needed on TFEB SUMOylation in cardiovascular diseases to address several unanswered questions: (1) Precisely how does SUMOylation regulate TFEB biological activity? (2) What upstream signaling molecules promote TFEB SUMOylation? (3) What roles do E1, E2, E3, and SUMO-specific proteases (SENP1, 2, 3, 5, 6, and 7) play? (4) How does TFEB SUMOylation alter the reverse cholesterol transport process and influence the occurrence and development of atherosclerosis? Answers to these questions may help identify TFEB SUMOylation sites as new therapeutic targets for atherosclerosis treatment.

Ischemic heart disease, characterized by stenosis or complete blockage of the coronary artery leading to insufficient blood supply and to myocardial cell damage, is one of the leading causes of death globally [69]. The current clinical treatment strategy aims to restore blood supply to ischemic tissue. However, ischemia/reperfusion (I/R) injury inevitably occurs during vascular reperfusion, exacerbating myocardial cell necrosis and causing cardiac dysfunction. Therefore, understanding the occurrence and regulation mechanism of myocardial I/R injury is crucial for identifying new therapeutic targets, protecting the myocardium, and improving clinical outcomes.

SUMOylation can modify various substrates in the cell, including critical subcellular organelles, to fine-tune cell survival and proliferation during heart development, as well as to regulate mitochondrial and sarcoplasmic reticulum function in physiological heart function [25]. Importantly, cardiac SUMOylation is being considered as a potential target for cardiovascular disease intervention and treatment. TFEB serves as a SUMOylation substrate and exhibited enhanced TFEB transcriptional activity in the myocardial nucleus, alleviating I/R injury by regulating autophagy homeostasis in the myocardial nucleus of middle-aged mice [27].

Additionally, analysis of cardiomyocyte survival rate under hypoxia/reoxygenation conditions revealed that downregulation of SUMO1 expression decreased cardiomyocyte survival rate and stress resistance, suggesting that SUMOylation-mediated regulation of TFEB activity is an important protective mechanism for enhancing the stress resistance of aging myocardia [27]. TFEB SUMOylation may alleviate post-ischemic injury in aging myocardia, improve cardiac function, and serve as a promising therapeutic target for the clinical prevention and treatment of ischemic heart disease in the elderly [27]. Conversely, ischemia leads to mitochondrial dysfunction, excessive production of reactive oxygen species (ROS), and calcium overload. These factors can inhibit deSUMOylation enzymes (SENPs) and activate specific E3 ligases, such as the protein inhibitor of activated STAT1 (PIAS1), thereby catalyzing the SUMOylation of TFEB, resulting in the obstruction of TFEB nuclear translocation and the suppression of its transcriptional activity and affecting the initiation of autophagy and lysosomal biogenesis [31,66,70,71]. In addition, damaged mitochondria accumulate, generating further ROS and promoting myocardial cell apoptosis or necrosis through pathways such as cytochrome c release. Moreover, impaired lysosomal function compromises the clearance of metabolic waste and the recovery of energy substrates, leading to an energy crisis and exacerbating the initiation and progression of ischemic heart disease [72,73]. Further studies are needed to elucidate the precise role of SUMOylation TFEB in heart muscle and blood vessels, its contribution to disease progression, and its relevant signaling pathways.

## 8. Conclusions and Future Perspective

**Figure 5:**
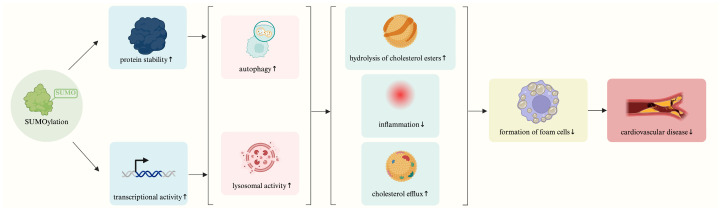
Schematic overview of the role of TFEB SUMOylation in cardiovascular disease. TFEB SUMOylation regulates the transcriptional activity and stability of TFEB, thereby modulating autophagy and lysosomal function, as well as cholesterol metabolism and inflammatory responses. These processes ultimately promote macrophage foam-cell formation and the development of cardiovascular disease. An upward-pointing arrow denotes upregulation; a downward-pointing arrow denotes downregulation. Created in BioRender (Xiaolong, C. (2025) https://app.biorender.com/illustrations/694246fdfaa555fc6f54aa82).

Recent advances have revealed that SUMOylation serves as an important post-translational regulatory mechanism for TFEB, influencing its stability, subcellular localization, transcriptional activity, and interactions with upstream signaling pathways. Through these mechanisms, TFEB SUMOylation modulates a broad spectrum of cellular processes, including autophagy and lysosomal function, lipid metabolism, inflammation, and foam cell formation, all of which are central to cardiovascular homeostasis and disease pathogenesis (Figure 5). Dysregulated TFEB SUMOylation has been observed in multiple cardiovascular disease models, such as atherosclerosis, myocardial ischemia, and metabolic cardiomyopathies, underscoring its emerging significance as a potential therapeutic target.

Despite these advances, substantial gaps remain. The upstream signals that dynamically regulate TFEB SUMOylation under physiological versus pathological conditions are still incompletely defined. In addition, the downstream gene programs specifically controlled by SUMOylated TFEB—and how these differ from those regulated by unmodified or other post-translationally modified TFEB—require clarification. Another critical unresolved question is whether TFEB SUMOylation exerts cell type–specific effects across cardiomyocyte subtypes, endothelial cells, vascular smooth muscle cells, macrophages, and other cardiac-resident immune cells. Furthermore, how TFEB SUMOylation interacts with other post-translational modifications, such as phosphorylation, acetylation, or ubiquitination, remains largely unexplored.

Addressing these knowledge gaps will be essential for determining whether TFEB SUMOylation can be selectively manipulated for therapeutic benefit. Future studies should aim to (1) map the upstream regulators and stress signals governing TFEB SUMOylation; (2) define the structural determinants and binding partners that mediate its functional outcomes; (3) delineate cell-type-specific roles in cardiovascular health and disease; and (4) evaluate pharmacologic or genetic approaches to modulate SUMO-TFEB signaling in vivo. Such efforts will deepen our understanding of TFEB SUMOylation across diverse cardiovascular conditions and may ultimately support the development of innovative therapies targeting this pathway.

## Data Availability

No new data were created or analyzed in this study. Data sharing is not applicable to this article.
